# Epidermal growth factor receptor (EGFR): A rising star in the era of precision medicine of lung cancer

**DOI:** 10.18632/oncotarget.16854

**Published:** 2017-04-05

**Authors:** Xiaomin Liu, Ping Wang, Caiyan Zhang, Zhongliang Ma

**Affiliations:** ^1^ Lab for Noncoding RNA and Cancer, School of Life Sciences, Shanghai University, 200444 Shanghai, China

**Keywords:** epidermal growth factor receptor (EGFR), noncoding RNA, precision medicine, lung cancer

## Abstract

Lung cancer is a leading cause of cancer mortality worldwide. In tumors, the important role of noncoding RNA regulatory networks has been more and more reveal. EGFR has been identified as an oncogenic driver of NSCLC, especially activating mutations EGFR and its inhibition with specific TKIs can generate dramatic tumor responses. Studies have shown that EGFR plays significant roles in the progression of NSCLC. Subset analysis of the small proportion of patients with EGFR-mutant lung cancer showed a disease-free survival benefit, but was underpowered to detect a survival advantage. Herein, we highlight the progression of EGFR, noncoding RNA, and their roles in carcinogenesis. We also focus on anti-lung cancer drug development and EGFR-related drug resistance.

## INTRODUCTION

Lung cancer is the leading cause of cancer-related mortality worldwide, and more than 1.5 million deaths is related with it every year [1]. The majority of patients present with locally advanced or metastatic disease. Approximately 85% of lung cancers are classified as non–small cell lung cancer (NSCLC), and include lung adenocarcinoma, squamous cell carcinoma (SCC), and large cell carcinoma (LCC) histologic subtypes. Over the past decade, major advances in the understanding of lung cancer, especially NSCLC, have been achieved [2]. NSCLC is defined as a group of different diseases, and as an oncogenic driver, epidermal growth factor receptor (EGFR) has been identified. Blockade of EGFR with specific tyrosine kinase inhibitors (TKIs) can generate dramatic tumor responses in NSCLC [3, 4].

EGFR is one of the four members of the HER family receptors, which compose of EGFR/HER1/erbB1, HER2/erbB2, HER3/erbB3, and HER4/erbB4. There are 11 species in the HER family of growth factors which can be broadly divided into those that specifically bind with EGFR (EGF, TGF-α, Amphiregulin (AR)), those that binding with EGFR and HER4 (BTC, HB-EGF, Epidermal regulators), and those that binding with HER3 and HER4 (Neuregulin). Although HER2 has no corresponding ligand, it usually binds to a ligand similar to the one that activates it, readily forming a dimer with other members of the HER family. Additionally, EGFR is a receptor of tyrosine kinase (RTK). It is consisted of a C-terminus intracellular region that possesses the kinase activity, and an N-terminus extracellular ligand-binding site, a hydrophobic transmembrane domain [5]. The EGFR signaling network plays a significant role in the epithelial tissues maintenance and growth, active EGFR signaling is frequently observed in lung cancer, and EGFR level is related with advanced stage of disease and bad prognosis [6]. As known, HER family receptor related malformation from primary lung tumors, NSCLC brain metastases have some strikingly differences [7]. Therefore, in the development of new drugs for cancer treatment, EGFR and its signaling components can be used as targets, such as chimeric monoclonal antibodies (panitumumab and cetuximab) [8] and TKIs (gefitinib, erlotinib, and afatinib) [9–11]. However, to date, cancer heterogeneity and the drug resistance greatly limit the usefulness of anti-EGFR agents [12].

**Figure 1:**
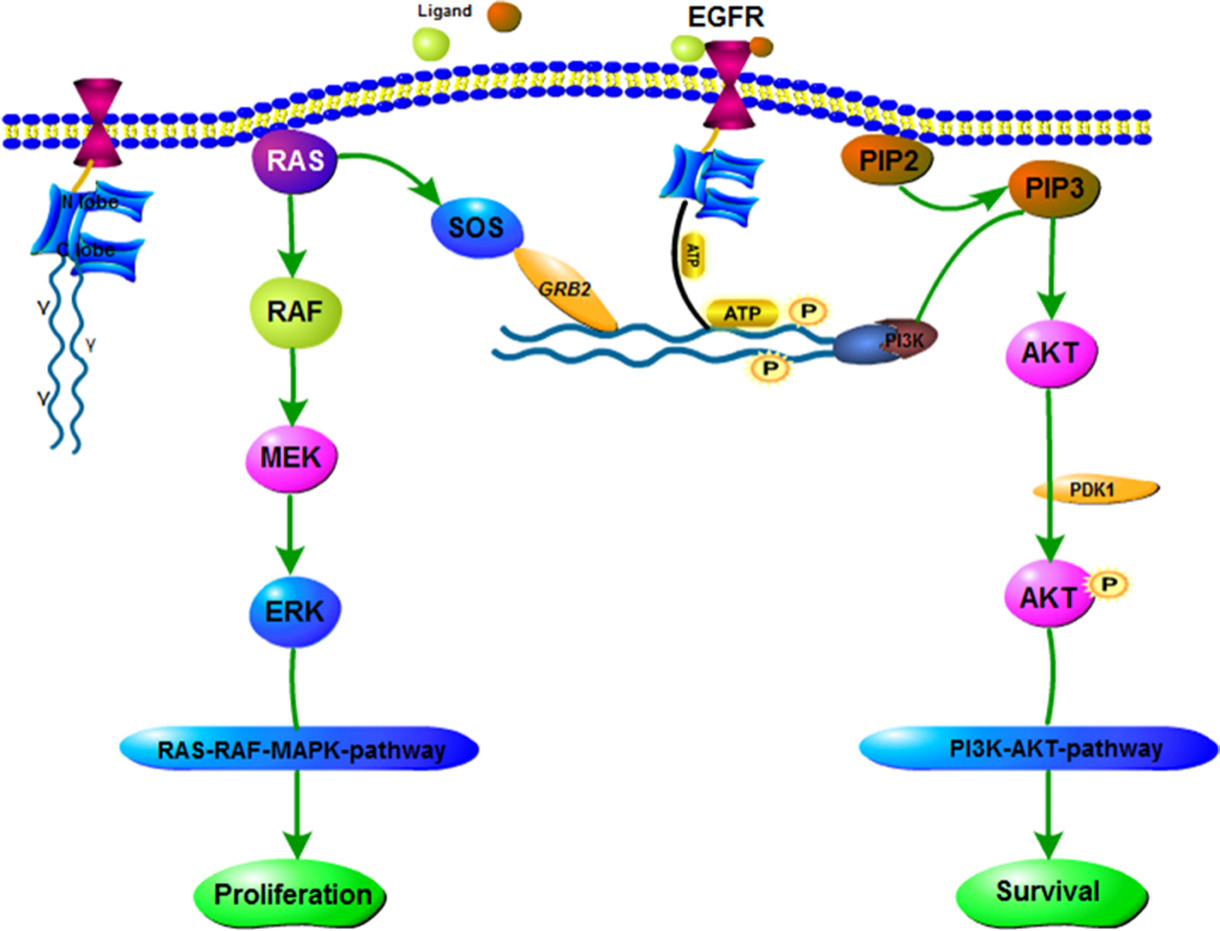
EGFR signaling pathway EGFR is a receptor protein that spans the cell membrane. TKI consists of N lobe and C lobe. EGFR-TKI competes with ATP for inhibition of this site. If the growth factor (ligand) binds to the receptor, it forms an asymmetric dimer. A variety of proteins associated with the phosphorylation of tyrosine, the downstream protein is constantly activated, as shown in chart the RAS-RAF-MAPK pathway and PI3K-AKT pathway.

EGFR gene is an oncogene-driven gene, tyrosine kinases (TK) active [13]. Meanwhile, discovered in 2004, EGFR mutation is the first molecular alteration in lung cancer that is shown to confer sensitivity to specific targeted therapies, namely TKIs [14–17]. EGFR-TKIs can inhibit EGFR autophosphorylation activation and its downstream signaling pathways through competitive binding with the EGFR binding region, preventing the binding of ATP and EGFR receptors. The EGFR signaling pathway is summarized in Figure 1.

These molecular alterations could be influential in SCC and have important implications in lung cancer treatment [18]. Currently, none of the recurrent molecular alterations, which are commonly altered in lung squamous cell carcinoma, have proven to be as predictive for response to therapy as EGFR alterations in lung adenocarcinoma. Despite the establishment of tumor profiling for lung adenocarcinomas, its clinical benefits for other histologic subtypes of lung cancer, such as lung SCC and SCLC, remain unclear. Furthermore, a significant minority of patients with NSCLC have activating mutations in EGFR [19], patients typically develop resistance within 9 to 12 months. Moreover, anti-EGFR antibody therapy uses extensive in treatment cancer [20].

In summary, studies have been focused on the underpinning mechanisms the resistance for anti-EGFR agents, and EGFR mutations play a pivotal role in lung cancer.

## EGFR AS A TARGET IN LUNG CANCER TREATMENT

Currently many studies have highlighted the relationship between EGFR and cancers. High expression of EGFR correlates with poor survival in cancer of the head and neck, as well as in cervical carcinoma and bladder [20–22]. In NSCLC, EGFR as a prognostic factor remains disputable. Some studies have verified that EGFR over-expression is predictive of a poor result in NSCLC [23–27], whereas others don't found such situation [27–29].

In the progression of NSCLC, EGFR, regarded as the cancer driver gene [30, 31], demonstrates the effects of numerous oncogenic, including stimulating DNA synthesis, cell cycle, cell proliferation, cell metastasis and invasion [32, 33], and has been proposed as an attractive and promising target for anti-cancer treatment [34]. The expression of high level of EGFR, the pathway can also be up-regulated by coexpression of receptor ligands (such as transforming growth factor-α (TGF-α) or EGF). Moreover, the duration of the EGFR signaling pathways are stimulated by the positive feedback loop formed by EGFR, a ligand-releasing protease and the RAS-MAPK signaling pathway (Figure 1). As the signaling response can be prolonged in a cell that is efficient in recapturing the endogenous ligand, in spite of the levels of EGFR expressed may be low [35]. Furthermore, a research in breast cancer verified that co-expression of TGF-α and EGFR had a more remarkable effect on survival, comparing to the co-expression of HER-2 and EGFR [36]. In lung cancer, one study has reported that synchronous expression of EGFR and HER-2 is predictive of increased recurrence risk. Moreover, the success therapy with programmed death ligand 1 (PD-L1) blockade in lung cancer suggests that immune escape mechanisms can be conducive to lung tumor etiopathogenesis [37].

## ACQUIRED RESISTANCE OF EGFR TKIS

In lung cancer, especially in NSCLC, a variety of EGFR mutations exist which are closely related to tumor development. The discovery of activating mutations in EGFR and the eventual approval of TKIs are the milestones in the history of NSCLC treatment.

### First-generation EGFR TKIs

**Figure 2:**
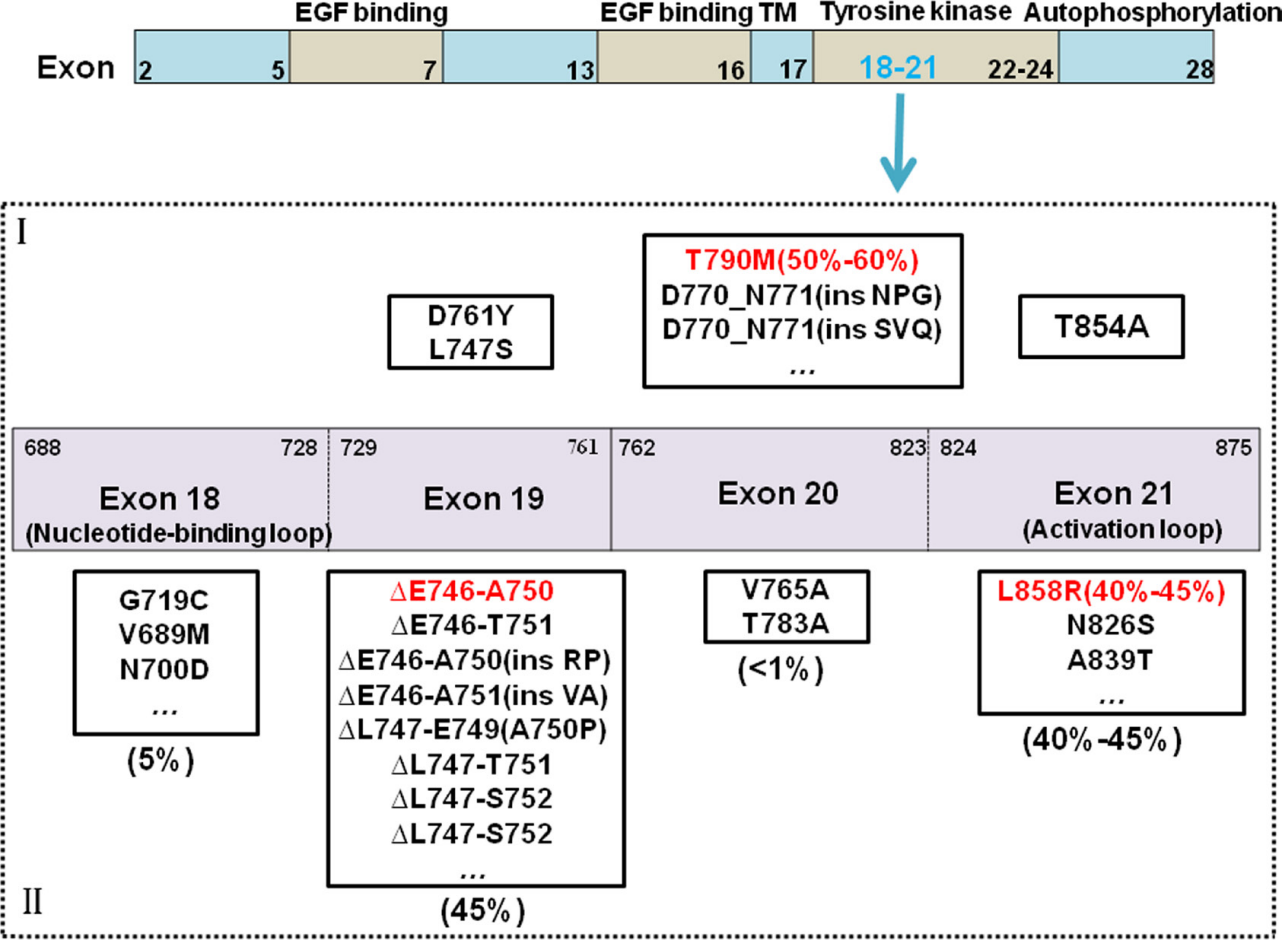
EGFR mutations and drug-resistant mechanism As common mutant sites, the mutations of exon18-21 in EGFR are discovered. It's including common mutations and rare mutations. Common mutations are involved deletion mutations in 45 percent of 19 exon, and point mutations of L858R in 40–45 percent of 21 exon. Others are rare mutations. The reason to raise drug-resistant is that it arises new mutations, the most important mutations is T790M in 50 percent. I stands for Mutations associated with drug resistant, II stands for Mutations associated with drug sensitivity.

Gefitinib and Erlotinib have greatly improved the progression-free survival over standard chemotherapy for EGFR-positive NSCLC. Yet, due to the emergence of resistance, disease progression eventually occurs in almost all patients. Quinazoline has the core skeleton of gefitinib and erlotinib combined with EGFR reversibility. However, almost all patients who received gefitinib/erlotinib treatment eventually and inevitably acquired resistance, with a median progression-free survival (mPFS) of approximately 9 to 11 months [38]. The most common mechanism of acquired resistance is point mutation of exon 20 to T790M, with an incidence of approximately 50% to 60% [39] (Figure 2). The threonine (T) at site 790 of EGFR is located at the ATP bound pocket of the tail end, and mutations in the kinase receptor-binding region form a huge chain methionine(M) residue, resulting in steric hindrance, which affects EGFR TKIs from binding to the receptor. Other mechanisms of resistance include MET gene amplification [40], small cell transformation [41], epithelial mesenchymal transition (EMT) [42], and so on.

### Second-generation EGFR TKIs

The second-generation EGFR TKIs were designed, in order to get around the problem of gefitinib and erlotinib resistance, and mainly included BIBW 2992 (afatinib), PF00299804 (dacomitinib), HKI-272 (neratinib), CI-1033(canertinib). The second-generation EGFR TKIs have greater potential to overcome or delay withstand to the first-generation EGFR TKIs [43], and have improvements in target, binding form, and efficacy. Sufficient evidences have not yet been accumulated to determine whether or not they can overcome the resistance to first-generation EGFR TKIs entirely. Recent discoveries based on these principles continue to inspire the next generation of innovative clinical trials in this diseases.

### Third-generation EGFR TKIs

To date, acquired resistance to EGFR-TKI is an unavoidable process and usually appears after 10–12 months of therapy. New EGFR-TKIs with specific capability bind to T790M mutated receptor have been developed and successfully tested in patients with acquired resistance [44, 45]. Moreover, the emerging third-generation EGFR TKIs have demonstrated high tolerability, through tested the higher ability to spare EGFR wild-type counterpart. The most famous of the third generation TKI is AZD9291 (osimertinib). It can response highly rate to drug-resistance in T790M mutations. Some patients showed resistance to this drug, and the major mutation site is C797S on the EGFR gene by the discovery of genome sequencing. The reason to drug resistance is including C-MET amplification, small cell lung cancer transformation, and downstream genes (KRAS or BRAF) activation. So patients can be controlled using a combination of the first and three generation EGFR-TKIs. With these evidences, AZD9291 (osimertinib), HM61713 (olmutinib), CO-1686 (rociletinib) and others (ASP8273, EGF816) are object of several clinical trials and AZD9291 has already obtained FDA and EMA approval for the therapy of EGFR mutant T790M-positive NSCLC [46].

In the future, when EGFR-TKI drug resistance occurs, genetic testing could be used to select the treatment method corresponding to the resistance mechanism.

## PRECISION MEDICINE OF EGFR IN LUNG CANCER

Precision medicine in the treatment of lung cancer has dramatically impacted diagnostic pathology, and precision medicine provides a better comprehension of both the mechanism of the disease at the molecular level.

**Figure 3:**
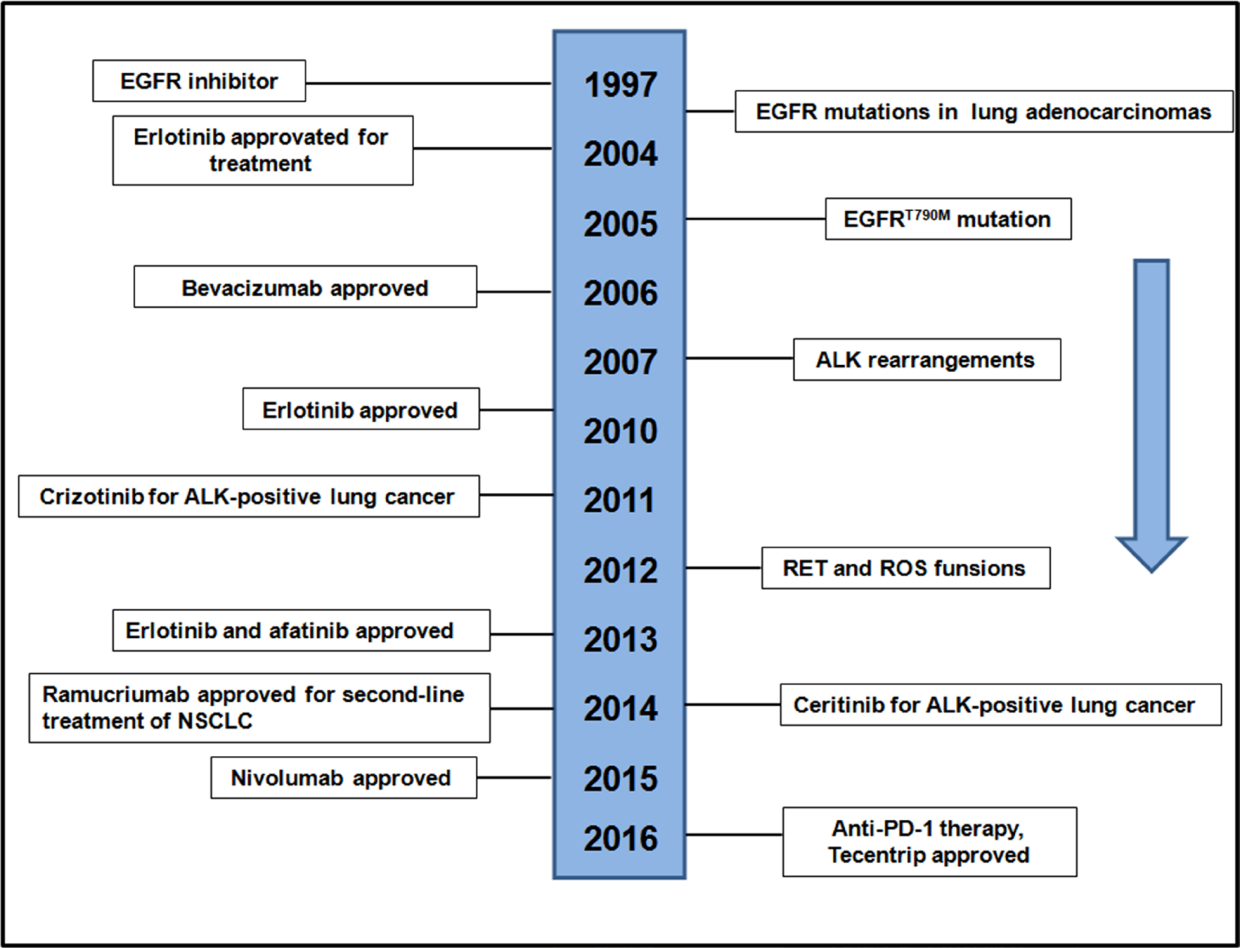
Timeline of EGFR-related drug development

The discovery of EGFR mutations and ALK-rearrangements were the first molecular alterations in lung adenocarcinoma that confer sensitivity to TKIs in 2004 and 2007 (Figure 3), heralding the initiation of the era of precision medicine for lung [15, 47]. Among EGFR mutations, EGFR^T790M^ mutation was a clear target for drug development to address the important precision medicine need, which identified as a mechanism of resistance to TKIs (Figure 3). The remarkable responses to TKIs observed in patients as well as the discoveries made studying these molecular subsets of lung cancer, served as catalysts for further exploration of the lung cancer genome, leading to the incorporation of molecular testing in routine clinical practice. Clinical trials have revealed that treatment of advanced EGFR lung cancers is superior to chemotherapy, using the appropriate TKIs [19, 48]. Conversely, it has also been shown that patients with the mutant EGFR lung cancers rarely respond to EGFR TKIs and are more likely to benefit from chemotherapy, underlining the importance of matching tumor genotype to therapy [48]. Here, we show the timeline of the history of EGFR development in recent years (Figure 3). At present and future efforts to find new types of precision medicine for lung cancers is necessary to improve outcomes for patients with lung cancer, as well as biomarker-driven clinical trials. In lung cancer, the impact of precision medicine has resulted in considerable changes as well as challenges in diagnostic pathology [49].

## NONCODING RNA AND EGFR

### MiRNAs in EGFR-targeted therapies for lung cancer

MicroRNAs (miRNAs) are a class of small noncoding RNAs that act as key post-transcriptional regulators of gene expression. They can functionally impact cell fate determination through the regulation of critical protein expression, thus playing a pivotal role in the diverse processes of human cancer, acting as either tumor oncogenes or suppressors [50–53]. Recently, increasing numbers of miRNAs have been correlated with the drug resistance of lung cancer cells to anti-EGFR agents, indicating that miRNAs may serve as novel targets or promising predictive biomarkers for anti-EGFR therapy. Moreover, miRNA-based therapy has been suggested to be a rational and potentially effective approach for the therapeutic targeting of EGFR [54]. Recently, a number of miRNAs, such as miR-200a, miR-27a/27b, miR-133a, and miR-134 have been verified to directly target EGFR [54–58] (Table 1). These EGFR-miRNAs regulation network studies demonstrated that miRNA-based therapy could possibly be utilized to target EGFR, except for TKIs and classical mAbs for EGFR-targeted therapies [54].

**Table 1:** miRNAs that target EGFR signaling pathway involved in cancer

miRNAs	Carcinomas	Biological effect	Regulation	Citations
miR-200	anaplastic thyroid cancer/bladder cancer	Regulate EMT and reverse resistance of EGFR therapy	Down	[65, 66]
miR-23b/27b	bladder cancer	Regulate EGFR and suppress cancer	Down	[55]
miR-27a	renal cell carcinoma	Suppress human RCC cell proliferation and induce cell apoptosis	Down	[56]
miR-133a	NSCLC	Suppresses multiple oncogenic membrane receptors and cell invasion	Down	[57]
miR-134	NSCLC	Inhibit proliferation	Down	[58]
miR-7	various cancer cells	Inhibit EGFR-PI3K-AKT signaling and reverse radio resistance	Down	[67]
miR-34a	solid cancer	Regulate Axl receptor tyrosine kinase by targeting SIRT1 and MEK1	Down	[68]
miR-145	lung cancer	Negatively regulate EGFR expression	Down	[69]
miR-146a	NSCLC	Inhibit EGFR in NSCLC cancer cells	Down	[70, 71]
miR-146b-5p	glioblastoma	Suppress EGFR expression	Down	[72]
miR-206	squamous lung cancer	Suppress EGFR signaling	Down	[73]
miR-135a-1	prostate cancer	Inhibit cell growth and migration	Down	[74]
miR-133a	NSCLC	Suppress EGFR signaling	Down	[57]
miR-133b	NSCLC	Suppress EGFR pathway signaling and enhance susceptibility to EGFR-TKI	Down	[75]
miR-1203,1237,541,542-5p	human lung cancer	Downregulate EGFR	Down	[67, 76]
miR-199a-3p	prostate cancer	Suppress the expansion and tumor	Down	[77]
miR-2861	cervical cancer	Inhibit tumor growth	Down	[78]
miR-25	lung cancer	Upregulate EGFR	Up	[79]
miR-24		Activates EGFR signaling	Up	[80]
miR-21	glioblastoma	Regulate the EGFR/AKT pathway in a PTEN independent manner	Up	[81]

Our lab has confirmed that miR-34a, miR-181a-5p, miR-32, and miR-486-5p play vital roles in the progression of NSCLC [59–61]. Our recent study demonstrated that miR-34a can suppress NSCLC by directly targeting EGFR *in vitro* and *in vivo* (paper in under-decision). These findings demonstrate that altered miRNA expression may be related to the oncogenesis of lung cancer. Some studies have verified that EGFR mutations can be regulated by miRNA in cancer therapies. At present study, small RNA possesses the best potential as a diagnostic biomarkers and therapeutic drug for cancer, which is the most mature miRNA. For instance, because of its pivotal role in lung cancer, liver cancer, breast cancer, and among others, miR-34a is considered to be the most likely of the miRNAs to become a diagnostic marker and target of drugs [62–64].

In conclusion, the emerging role of miRNAs as regulators could not only active EGFR signaling, but also the lung cancer cells resistance to anti-EGFR therapy. Additionally, miRNAs could also be employed as novel therapeutic targets to circumvent the resistance of lung cancer cells to EGFR inhibitors, and as biomarkers for response to anti-EGFR agents.

### LncRNAs in EGFR- TKIs for lung cancer

Long non-coding RNAs (lncRNAs) are non-coding RNAs with a length > 200 nt. It has been revealed that lncRNAs are involved in a number of biological processes, such as chromatin modification, gene regulation, transcription activation and interference, and cellular processes, including cell apoptosis, migration, tumor invasion, metastasis, and drug resistance [82–84]. They play vital roles in the tumor incidence and development.

**Figure 4:**
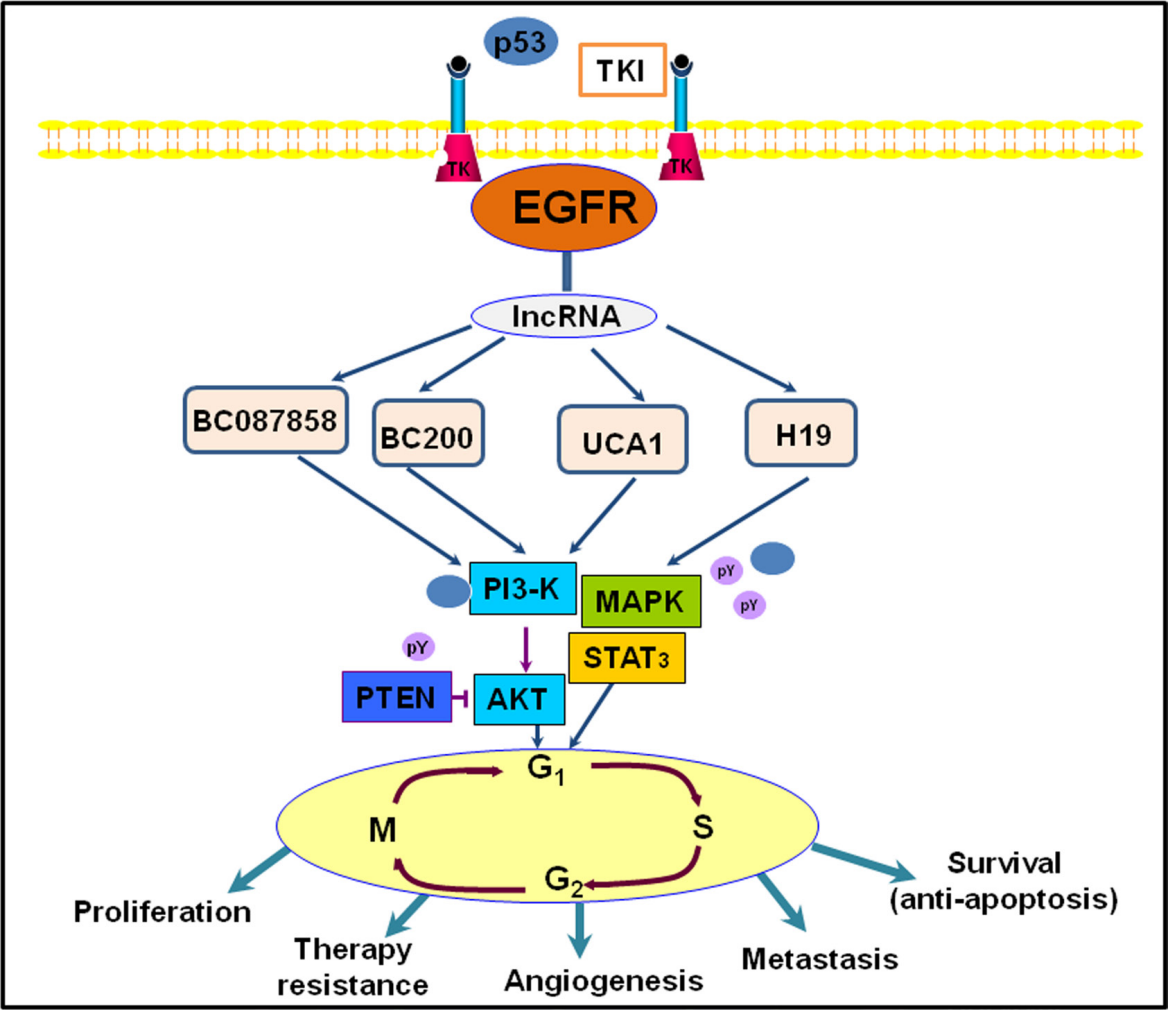
An illustration representing long noncoding RNAs (lncRNAs) and they involved in EGFR signaling pathway in lung cancer

Currently study shown that lncRNAs can connect to transcription sites and regulate both the expression of alleles and a long fragment, whereas coding genes and miRNAs have no such functions [85]. This suggests that lncRNAs may be better epigenetic regulators in gene expression regulation. Research has shown that some lncRNAs, including lncRNA UCA1, H19, BC200, and BC087858, were increased in gefitinib-resistant human lung cancer cells, as determined by lncRNA microarray analysis [86]. Studies have also demonstrated that lncRNA UCA1 may stimulate non-T790M acquired resistance for EGFR-TKIs by activating the AKT/mTOR pathway and EMT [87]. Moreover, over-expression of lncRNA BC087858 could act via a novel mechanism by which acquired resistance for EGFR-TKIs can develop in EGFR-mutant NSCLC patients without T790M mutation [88]. Another study showed that stimulation of the PI3K/AKT and MEK/ERK pathways, as well as EMT, could be implicated in the resistance to EGFR-TKIs [89, 90]. Herein, we illustrate that lncRNAs are involved in the EGFR signaling pathway, as displayed in Figure 4.

In conclusion, further exploration of the function and mechanism of lncRNAs will reveal their critical role in the process of lung cancer generation, and their significance as a diagnostic tool as well as in the treatment of cancer.

### tRNA-derived RNA fragments and EGFR

A close connection has been established between cancers and a variety of small noncoding RNAs, such as miRNAs, piRNAs, and circRNAs, but not including tRNA-derived RNA fragments (tRFs). tRFs, the class of small RNAs, are noncoding-stranded RNAs 14–35 nt in length, which always derive from the 5′ end or 3′ end of tRNA in the particular environment [91]. The length and the generation of tRFs are very similar to those of miRNAs. Through the action of anticodon-cleaving enzymes, some tRNA cleavages can be generated from mature tRNAs. The biological function of tRFs is still unclear. Some studies have shown that, in biological processes, tRFs have the capacity to regulate some cellular processes, including translational efficiency under stress conditions [92], oncogenic transformation [93], and mitochondrial-mediated apoptosis [94]. EGFR and transferrin receptors (TFR) are known to be involved in cell growth and to be expressed in normal human epidermis. Currently, about EGFR and TRF expression in human cancer is few.

However, their biological function has drawn the attention of many research efforts. Some studies have shown that, in biological processes, the function of 5' tRFs might extend much farther than that of 3' tRFs [95, 96]. In addition, it has been found that tRFs suppress breast cancer progression via YBX1 displacement [97]. Furthermore, some tRFs can active cellular functions through argonaute engagement, such as cell proliferation and RNA silencing [98–100]. TRF expression is not associated with cellular proliferation, in embryonic and fetal epidermis, whereas EGFR appears to be correlated with proliferation and undifferentiated cells [101].

## PERSPECTIVES AND CHALLENGE IN EGFR-RELATED STUDY

The evolution of modern medical technology has enabled advancements in research to reveal the mechanism of the development of a variety of incurable disease. In the case of cancer, the emerging view is that cancer is a “genetic disease”. With next generation sequencing technology and promotion of the human genome project, the treatment of cancer has been gradually moving towards the era of precision therapy. Under this condition, new therapies take advantage of RNA and other powerful features targeting genes that play a critical role in tumorigenesis.

The importance of miRNA regulatory network's role in tumors has been revealed more and more. Meanwhile, we should combine molecular mechanisms to overcome the emergence of resistance. As described in this review, several molecular pathways underlying the mechanisms of this disease have been elaborated in part, among them the EGFR pathway is one of the well-known signal cascades that play a pivotal role in oncogenesis. In lung cancer, dysregulation of EGFR signaling is frequently found. Strategies to effectively inhibit the EGFR signaling pathway have been mounted for developing anticancer therapeutic agents. However, on account of the development of drug-resistance, most anti-EGFR-targeted agents are unable to repress cancer progression. Therefore, studies of the mechanisms underpinning the resistance toward anti-EGFR agents may afford important findings for the use of anti-EGFR therapies in lung cancer treatment.

Finally, when determining the applicability of EGFR-TKI in practice, clinical benefit should be carefully analyzed based on clinical background and the prediction of the presence or absence of EGFR mutations. Furthermore, immune-based cancer prevention can also influence premalignant biology. It has been shown that cancer vaccines reprogram the immune response to prevent, reject, and detect premalignant cells, which might be applicable in EGFR therapy [102]. Going forward, in lung cancer, EGFR may be the rising star in the era of precision medicine.
